# AI-quantum framework for accurate infertility risk classification in PCOS patients using EHR data

**DOI:** 10.3389/frai.2026.1776461

**Published:** 2026-03-24

**Authors:** T. Sarath, K. Brindha

**Affiliations:** 1 School of Computer Science Engineering and Information Systems, Vellore Institute of Technology, Vellore, Tamil Nadu, India; 2 School of Computer Science Engineering and Information Systems, Vellore Institute of Technology, Vellore, Tamil Nadu, India

**Keywords:** artificial intelligence, electronic health record, infertility, LSTM, polycystic ovary syndrome, quantum computing

## Abstract

**Introduction:**

Infertility affects nearly one in six couples worldwide, with approximately half of these cases associated with female-related factors. Among women, ovulatory disorders represent a major cause of infertility, with Polycystic Ovary Syndrome (PCOS) being one of the most prevalent conditions. Traditional diagnostic methods, including ultrasound imaging, hormone analysis, and menstrual history evaluation, often fail to fully utilize comprehensive patient health records. These records contain important clinical indicators such as body mass index (BMI), Anti-Müllerian Hormone (AMH) levels, follicle count, and menstrual irregularities, which may provide deeper insights into infertility risk.

**Methods:**

This study proposes a novel AI–quantum hybrid framework for analyzing PCOS-related health records to improve infertility risk prediction. The framework integrates classical deep learning techniques with quantum-inspired models, including Long Short-Term Memory (LSTM) and Quantum LSTM architectures, to capture long-term dependencies in sequential clinical data. The proposed models are trained and evaluated using a PCOS dataset containing multiple clinical attributes relevant to reproductive health.

**Results:**

The experimental analysis demonstrates that the hybrid AI–quantum framework effectively identifies complex relationships within clinical data and improves infertility risk classification compared with traditional machine learning approaches. The model shows enhanced capability in handling heterogeneous and incomplete health records while maintaining robust predictive performance.

**Discussion and conclusion:**

The findings highlight the potential of integrating artificial intelligence with quantum-inspired computing techniques to support early detection of infertility risks associated with PCOS. The proposed framework can assist clinicians in identifying high-risk patients more efficiently and may contribute to personalized reproductive healthcare and improved diagnostic accuracy.

## 1 Introduction

Infertility is a major health problem that affects people physically, emotionally, and financially. Globally, millions of women are most affected by infertility, mainly due to ovulation disorders commonly known as polycystic ovary syndrome (PCOS). This condition disrupts hormonal balance in women, causing infertility and a reduced quality of life (Christ and Cedars, 2023). To enhance reproductive outcomes and improve quality of life, early diagnosis and personalized therapy are important.

For personalized therapy, analyzing electronic health records (EHRs) is one of the best ways to achieve early disease diagnosis. EHRs are long-term patient data collections that includes medical history, test results, lifestyle information, and image investigations. The analysis of EHRs provides a strong foundation for identifying patterns and data-driven plans to understand the causes of diseases. Combining computational tools with health record data enhances risk stratification and enables early intervention measures (Attia et al., 2023) through predictive modeling. However, diagnosing PCOS using health records remains challenging for clinics to perform this task quickly and accurately.

Analyzing health record data using artificial intelligence (AI) and quantum computing techniques provides a promising solution, as these methods can enhance clinical decisions, personalize patient care, and assist clinicians in treatment planning. However, the models provide effective treatments and clinical explanations to overcome complex treatment challenges, making improved treatment methods easier to understand and apply.

Therefore, the main goal of this study is to deliver insights that improve diagnosis accuracy, promote early detection, and support clinicians with evidence-based visual tools by examining the predictive value of numerous physical, hormonal, and lifestyle characteristics associated with PCOS using a novel AI–Quantum framework with several LSTM and quantum LSTM models.

This study begins with an extensive literature review on the topic and then describes the approach used to examine the challenges of infertility using healthcare data pertaining to women. Following an explanation of the methodology, dataset properties, and experimental analysis, the report concludes with a discussion on the most important results and conclusions.

## 2 Related works

EHRs enable the systematic collection of comprehensive patient data across multiple healthcare institutions over extended periods of time. Many researchers have demonstrated that EHR-based analytics can uncover latent patterns associated with disease onset, progression, and comorbidities, thereby enabling early risk stratification and improved clinical outcomes. However, capturing and predicting these patterns using traditional models remains challenging due to high dimensionality, heterogeneity, and temporal nature of EHR data. As a result, deep learning (DL) models are increasingly used to identify underdiagnosed conditions and to aid in preventive and personalized healthcare.

EHR data related to PCOS provide a valuable resource for identifying infertility in women. PCOS-related EHRs offer a holistic view of reproductive, metabolic, and hormonal health, making them a valuable resource for early diagnosis and long-term risk prediction.

Aggarwal and Pandey (2023a) addressed this question by identifying commonly known diseases, including obesity, diabetes, heart disease, and high blood pressure, as indicators of PCOS. To achieve this, diabetes and heart disease datasets were taken, and both the supervised and unsupervised learning algorithms were applied. The results concluded that 50% of women affected with PCOS had a chance of developing obesity and diabetes, and were highly prone to heart diseases.

Identifying vital signs and clinical characteristics of PCOS based on health records using AI-based algorithms is one of the most used technique by researchers. These analyses can be performed by developing models for feature selection to accurately diagnose the disease and evaluate medical approaches using large amounts of PCOS-related health record data (Rahman et al., 2023).

Lim et al. (2023) used ML methods for forecasting and sorting PCOS cases with pulse wave parameters in traditional Chinese medicine. For this purpose, 459 patient data were used with the voting model and the long short-term memory (LSTM) model. The LSTM model was found to be the best, achieving an accuracy of 0.72 accuracy and AUC values of 0.715 and 0.722. Based on this analysis, the author explored PCOS and methods for early-stage treatment using radial pulse wave data.

Yamini et al. (2023) applied several machine learning (ML) models for predicting PCOS using clinical, hormonal, and biomarker data. The study’s dataset included both PCOS-afflicted and non-afflicted women. The study used a number of ML methods, such as logistic regression (LR), XGBoost, Knowledge-Based Neural Network (KBNN), Support Vector Machine (SVM), Naïve Bayes (NB) classifier, and Random Forest (RF) to build its prediction models. With an accuracy rate of 90%, the RF model stood out among the other models examined. Na et al. (2022) used data from the International Gene Expression Omnibus to investigate immune infiltration as a correlate of functional PCOS biomarkers. This study identified two biomarkers in PCOS pathogenesis, as demonstrated by their high correlation with invading immune cell types. Table 1 summarizes some of the findings from many studies that examined PCOS using EHR data with AI models.

**Table 1:** Summary of findings from researchers.

Authors	Algorithms used	Data source used	Accuracy
Harerimana et al. (2019)	DL	Large-scale EHR	Provided insights into how EHR using DL approaches can be applied for clinical knowledge discovery. The study discusses technical side of various efforts that invested to apply DL to support health informatics and professionals in extracting features form vast EHR datasets.
Aggarwal and Pandey (2022)	Design of experiments (DOE), 2^K-p^ fractional design, validation with ANOVA and Minitab	PCOS dataset containing 541 instances and 7 clinical attributes	Demonstrated PCOS can be effectively identified using fewer parameters. Reduced seven diagnostic parameters to four significant predictors, including follicle number (right), FSH/LH ratio, follicle number (left), and skin darkening. Identified 28.44, 21.36, 15.29, and 15.29% contributions conforming their statistical significance for eliminating variables in PCOS-related studies.
Khanna et al. (2023)	Naive Bayes (NB), Decision Tree (DT), K- Nearest Neighborhood (KNN), Random Forest (RF), Support Vector Machine (SVM), AdaBoost (AB), Extra Trees, and Gradient Boost.	Survey conducted among 541 women from Kerala.	The findings showed best outcomes with an accuracy of 98%. However, the use of multiple models on a single dataset may lead to overfitting, which is one of the study’s limitations.
Wagh et al. (2021)	Various ML techniques, Principal Component Analysis (PCA)	Survey conducted among 541 women	Most accurate: RFC at 89.02% Employed statistical tools and PCA for feature reduction.
Bhat (2021)	RF, LIME	PCOS dataset available online	Accuracy: 86.03%, Sensitivity: 86.32%, Specificity: 85.37% Identified critical features influencing the presence or absence of PCOS, enhancing understanding of key diagnostic indicators.
Aggarwal and Pandey (2021)	SVM, LR, GB, RF, DT, K-NN	PCOS dataset containing 541 instances and 41 attributes	After applying feature extraction, from 41 attributes 12 attributes are identified for diagnosing PCOS and concluded identified features are not sufficient for PCOS diagnosis.
Aggarwal and Pandey (2023b)	Simple classification: SVM, LR, GB, RF, DT, K-NN and hybrid algorithms: RFGB, DTGB, KNNGB, LRGB, SVMGB.	Taken two datasets: Heart disease and diabetes	simple classification GB (98.4%) got high accuracy and in Hybrid, LGRB (98.4%) is best along with less time for execution.
Danaei Mehr and Polat (2022)	Non-invasive and invasive models	Not specified	Examined efficacy of both non-invasive and invasive predictors in PCOS diagnosis, emphasizing the importance of various clinical signs and metabolic parameters in enhancing predictive accuracy 81–90.1%
Pandey et al. (2023)	Extra tree classifier methods: SVC, LR, RF, DT, KN, Stacked Model, XGBRF, CatBoost with forward feature selection, chi-square test.	Kaggle PCOS Dataset	CatBoost classifier is the best algorithm with 0.97% accuracy and assists the user in predicting PCOS at early stage.
Zad et al. (2024)	RFs, Gradient-Boosted Trees (GBTs), SVMs, and Linear Regression (LR)	30,601 (EHRs)	AUCs ranging from 77.4 to 85%, with GBT being the best performer.
Adla et al. (2021)	Linear SVM	Dataset of 541 patients from Kaggle.	Accuracy: 91.60%
Silva et al. (2022)	BorutaShap, RF	73 Healthy women and 72 PCOS patients	Accuracy: 86.00%
Zigarelli et al. (2022)	CATBOOST	Dataset of 541 patients from Kaggle.	Accuracy: 90.10%
Panjwani et al. (2025)	Ensemble learning (RF and SHAP) with different optimizers such as Walrus, Cuckoo, and Random search	Two public related dataset merged into symptomatic dataset with 12 search features.	Walrus optimizer with RF and SHAP ensemble model got the high accuracy with 92.8% and 0.93 AUC.
Rahman et al. (2024)	LR, DT, AB, RF, SVM	Dataset of 541 Patients from Kaggle.	AB and RF achieved highest accuracy with 94%
Khan et al. (2024)	Quantum Long Short-Term Memory (QLSTM), compared with classical LSTM	Real-world photovoltaic dataset	QLSTM achieved fastest training convergence lower test losses and improved generalization compared to classical LSTM.
Wu and Zhang (2025)	Quantum Convolutional Neural Network including classical models: SVM, XGBoost, LightGBM. CatBoost, Multilayer Perception	Publicly available Kaggle ultrasound images of PCOS dataset	CatBoost and LightGBM suppressing benchmark with 98 and 100% test accuracy.
Tripathi et al. (2025)	Quantum Long Short-Term Memory (QLSTM), VQC, Efficient Ansatz Design	Malware system call sequences dataset	Quantum machine learning demonstrated improved data analysis with optimizing circuit and qubit count.

There is an increasing interest in investigating the methodologies and applications of quantum computing within the medical sciences (Zhao et al., 2018). Rasool et al. (2023) examined healthcare from the perspective of quantum computing and discussed about current trends. Mensa et al. (2023) explored how quantum computing could be used in computational biology, highlighting the limitations of current quantum hardware and the possibilities of the emerging field of quantum computational biology. Flöther (2023) devised a methodology employing a deep reinforcement learning framework for clinical decision support, leveraging the Qiskit quantum computing simulator and IBM quantum computers. Their research seeks to enhance individualized patient dosage by integrating patient-specific data, encompassing biological, physical, genetic, clinical, and diametric elements pertinent to radiotherapy. This study centers on optimization, data processing, simulation, and healthcare.

Botsinis et al. (2019) investigates quantum search algorithms for wireless communication, whereas Anastasova et al. (2021) elucidates the computational constraints of classical computing devices and describes solutions based on quantum superposition and entanglement. The authors (Maniscalco et al., 2022) specifically built the Quantum Dot Plot (QDP) to address inadequacies in classical data encoding techniques, taking into account the exponential speedups offered by the Quantum Pairwise Sequence Alignment (QPSA) algorithm. They implemented QDP and QPSA work on IBM’s superconducting architecture and AQT’s trapped ion architecture, which works with universal gate machines.

There is already substantial research on using AI to diagnose and treat PCOS, but there are still a lot of key areas that might be improved upon to make diagnosis even easier and treatment even better. To begin with, most recent research on the usefulness of different AI models for PCOS prediction relies on simulations rather than real-world data. Furthermore, the integration of quantum inspired computational techniques in PCOS research is still at a nascent stage with minimal translation into clinical practice. This means that although several AI–quantum approaches have proven to be effective in predicting PCOS, their use in clinical practice is still minimal. It is also determined that in order to improve the prediction models’ usefulness, more data with higher variability, particularly regarding demographic and genetic traits, needs to be generated. Therefore, there is a clear need for advanced predictive frameworks that improve diagnostic accuracy while ensuring clinical feasibility.

## 3 Methodology

**Figure 1:**
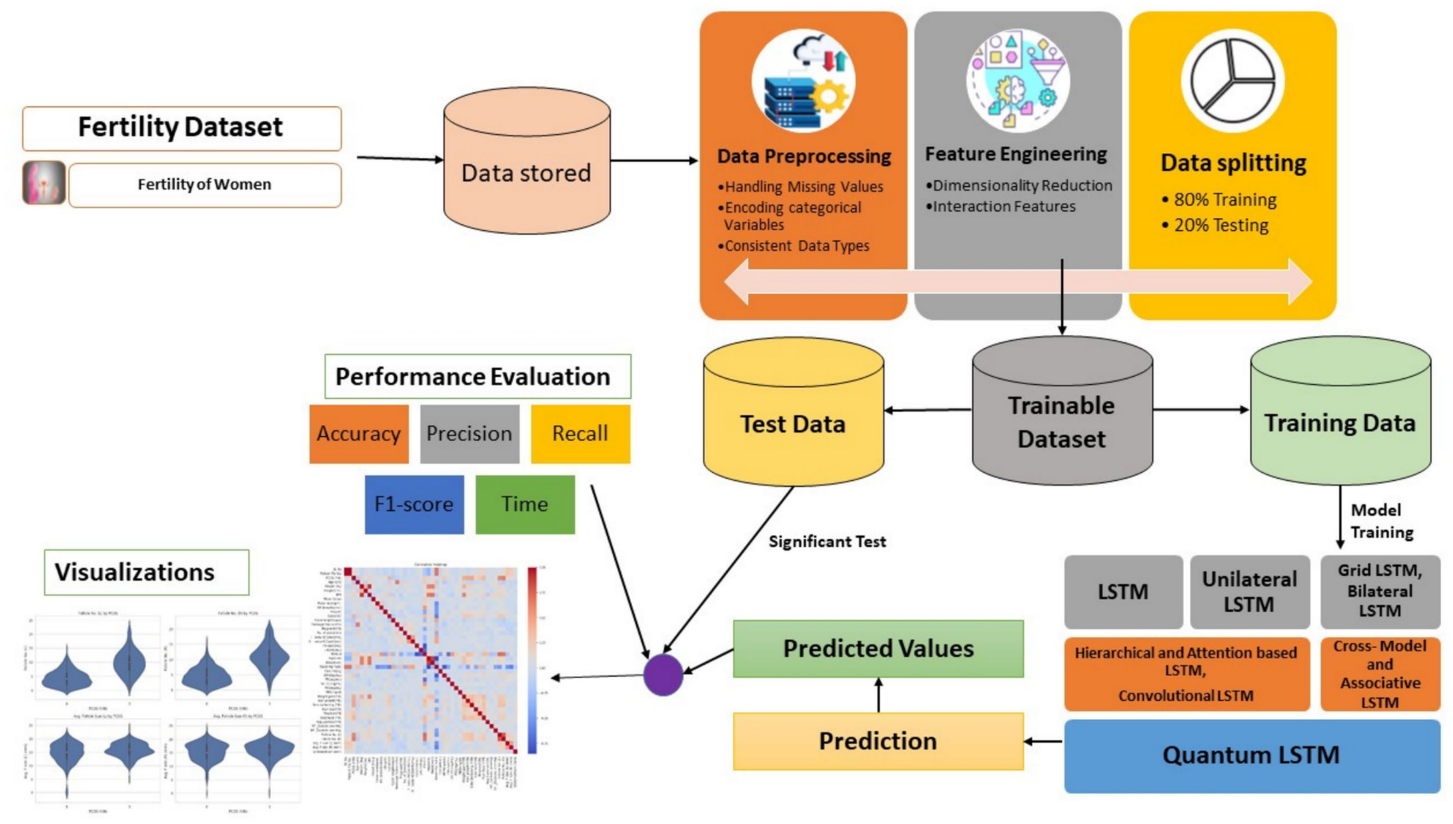
Step-by-step prediction procedure.

This study focuses on expediting patient diagnoses by creating a model to monitor and improve patient care for infertility in women, based on female patient activity record data related to PCOS. For this purpose, a novel AI–Quantum framework with various LSTM and quantum LSTM models are applied on the dataset. The step-by-step prediction procedure is depicted in Figure 1.

### 3.1 Methods

This research proposes AI–Quantum frame work for predicting infertility in women based on PCOS EHR data analysis. The pipeline integrates data pre-processing, feature engineering and data splitting followed by training of various LSTM and a proposed Quantum LSTM models to predict and are evaluated using standard performance metrics and visual analytics to support interpretability and clinical insights. The description of various applied LSTM models are as follows:

#### 3.1.1 LSTM

A unique design is used by LSTM recurrent neural networks (RNNs) (Britz et al., 2017) to simulate sequential data and capture long-term dependencies. For tasks involving long sequences, LSTMs outperform standard RNNs due to their ability to solve the vanishing gradient problem.

#### 3.1.2 Unilateral LSTM

In the same way as traditional LSTM networks can only process data in one direction (Britz et al., 2017), typically from the past to the future, unilateral LSTMs can only process data in one direction. This method finds use in anomaly identification, time series forecasting, and similar sequential data jobs that depend on previous information for prediction (Cui et al., 2018).

#### 3.1.3 Bidirectional LSTM

To handle sequence in both directions, bidirectional long short-term memory (BiLSTM) is used, which is a one of the form of LSTM (Cui et al., 2018). The BiLSTM is a best choice for predicting present and future facts for visualization (Ma et al., 2018) and it differs from Unilateral LSTMs.

#### 3.1.4 Hierarchical LSTM and attention-based LSTM

Understand challenges in complicated sequence modeling is difficult in normal LSTM between interconnections and interdependencies (Xue et al., 2018). To address this, attention-based and hierarchical LSTMs can be used.

##### 3.1.4.1 Hierarchical LSTM

Hierarchical LSTM (Su and Jiang, 2020) is a tree like structure to process sequential input because it breaks large sequence into smaller parts using lower level LSTMs and combines output using high level LSTM.

##### 3.1.4.2 Attention-based LSTM

Attention based LSTM works by distributing sequence data to different levels of weight (Villegas et al., 2017) at each time step. Instead of assigning the same weight, this method distributes greater weight to essential forecasts.

#### 3.1.5 Convolutional LSTM

Deals with spatial (images and video frames) and temporal (time series) data Convolutional Long Short-Term Memory (ConvLSTM) (Chu et al., 2019; Huang and Kuo, 2018) is best method. In regular LSTM layers are fully connected whereas in ConvLSTM designed with convolutional layers to differentiate patterns and correlations in spatial and temporal data (Kim and Cho, 2019).

#### 3.1.6 LSTM auto encoder

In LSTM auto encoders, sequence of data are encoded and decoded with LSTM networks (Gensler et al., 2016). Common uses include reducing data size, extracting features, finding outliers, and reconstructing sequences. A decoder uses a compressed, low-dimensional representation of an input sequence to reassemble the original sequence (Lindemann et al., 2019). It is likely that the input data is not normal, if the reconstruction error is substantial (Hsu, 2017).

#### 3.1.7 Grid LSTM

Using a grid framework to manage data with several dimensions, Grid LSTM is an upgraded form of LSTM (Kalchbrenner et al., 2015). While traditional LSTMs operate in a single temporal dimension, Grid LSTMs can simultaneously analyze data along multiple dimensions (Cheng et al., 2018).

#### 3.1.8 Cross-modal and associative LSTM

Additional specialized designs, such Cross-Modal and Associative LSTMs, enhance the ordinary LSTM for analyzing and correlating multi-modal data or executing associative learning projects (Veličković et al., 2018).

##### 3.1.8.1 Cross model LSTM

Cross-Modal LSTM can model diverse data types, including text, audio, video, and sensor data. Additional tasks that gain a lot from this method include video captioning and emotion recognition (Tay et al., 2017), both of which necessitate understanding the interaction between several types of input. Hence, LSTM networks pool data from many modalities to obtain the joint representation.

##### 3.1.8.2 Associative LSTM

Because they form associations between various patterns or sequences, associative LSTMs are able to capture sequences of interaction between various sequences (Danihelka et al., 2016). Taking its cue from how humans recall things, it uses that data to infer the relationships between future events.

#### 3.1.9 Quantum LSTM

Quantum computing uses qubits to leverage the principles of superposition and entanglement, enabling computation of vast amounts of complicated data simultaneously, which is not possible in classical computing. The Hadamard gate generates superposition states, and Variational Quantum Circuits (VQC) (Khan et al., 2024) combine quantum processes with classical optimization to perform learning tasks rapidly.

A QLSTM network builds on classical LSTM by putting sequential data into quantum states and processing them with parameterized quantum circuits. LSTM-like gating combines expectation values from quantum measurements with hidden states, which makes state representations more complex.

The QLSTM structure has:

- Quantum embedding of classical inputs
- VQC-based memory cell for sequential processing
- Classical–quantum interface for encoding and decoding
- Layer for making predictions based on feedforward output.

QLSTM uses quantum parallelism to accelerate convergence, handle noisy data more affetively, and identify patterns in healthcare datasets. This makes the method very useful for early diagnosis of complex illnesses like infertility due to PCOS.

### 3.2 Dataset

This dataset, which focuses on PCOS-related infertility,^1^ comes from the Kaggle repository. This cohort exhibits symptoms such as abnormally long or irregular menstrual periods and high testosterone levels. Problems with egg production, known as PCOS-related infertility, can arise from the development of small fluid-filled collections called follicles in the ovaries. There are no missing clinical or physical markers for PCOS in this sample. The data were submitted by 10 medical facilities located in the Indian state of Kerala. The datasets contain 44 parameters: some are binary (yes/no, denoted by 1 and 0), while others are represented by their respective values.

Here, the classification of patients into PCOS as 0 and Non-PCOS as 1 category is derived directly from feature distributions present in dataset predefined diagnostic cutoffs. Let each patient represented by feature vector:

Y={F,H,M,A,L}

where F denotes morphology features of ovary, H signifies hormonal makers, M denotes menstrual characteristics, A denotes anthropometric and clinical features, and L signifies lifestyle factors.

From the dataset analysis, PCOS-positive patients exhibit higher frequency and stronger deviations in a subset of these features. The implicit constraint learned by model is expressed as,

$$\text{PCOS}=1\;\text{if}\;\sum_{j=1}^{n}\prod (X_{j}\in P_{\text{PCOS}})\geq T$$

where $P_{\text{PCOS}}$ denotes feature patterns commonly observed among PCOS-positive patients, ∏() is function indicator, and T signifies decision determined during training.

## 4 Experimental analysis

Data collection was followed by a comprehensive pre-processing workflow to ensure quality and reliability of the dataset. Data cleaning was performed as an initial step to eliminate duplicate entries, incorrect or missing values, and any inconsistencies or anomalies present in the records. This step ensured dataset was accurate, complete, and suitable for model training.

Subsequently, feature engineering was applied to select, transform, and construct relevant features that enable models to effectively capture underlying patterns associated with PCOS, such as ovarian follicle characteristics, hormonal imbalances, and metabolic issues. These engineered features represent key physical, metabolic, and reproductive symptoms relevant to PCOS diagnosis.

After pre-processing and feature engineering, the required Python libraries were installed and configured for model development. Various LSTM-based architectures were then employed for training, as these models are well-suited for capturing complex dependencies and interactions among features. The dataset was divided into 80% for training and 20% for testing, allowing for an unbiased evaluation of model performance across various LSTM variants.

To address the multivariate structure of dataset, various LSTM models were employed to determine the most suitable architecture for given data conditions. A diverse set of LSTM variants was explored, including LSTM, Unilateral LSTM, Bidirectional LSTM, Hierarchical and Attention-based LSTM, Convolutional LSTM, LSTM Autoencoder, Grid LSTM, Cross-Modal and Associative LSTM, and quantum LSTM.

Each model is designed to handle complex, heterogeneous, and sensitive temporal data in a unique manner. The Standard LSTM leverages gated mechanisms (input, forget, and output gates) to effectively capture long-term dependencies while mitigating the vanishing gradient problem; Unilateral LSTM processes information in a single temporal direction, making it suitable for tasks that rely solely on historical data; Bidirectional LSTM analyzes sequences in both forward and backward directions, enabling incorporation of contextual information from past and future time steps; Hierarchical and Attention-based LSTM architectures manage multi-level temporal representations and dynamically emphasize most informative parts of input sequence; Convolutional LSTM integrates convolutional operations with temporal modeling, allowing it to capture both spatial and temporal correlations present in structured health data; LSTM Autoencoder learns compact latent representations of sequential data and reconstructs the input sequence, making it effective for feature learning and anomaly detection; Grid LSTM extends traditional LSTM processing across multiple dimensions, enabling the analysis of more complex data structures; and Cross-Modal and Associative LSTM architecture capture interdependencies across different data modalities by learning associations among clinical, hormonal, and lifestyle feature streams.

The quantum LSTM was implemented in Python using the PennyLane quantum simulator, which enables the integration of parameterized quantum circuits with classical ML frameworks. In this architecture, quantum layers composed of qubits and quantum gates replace selected neural components of classical LSTM, allowing quantum classical interaction during training. The model was developed using PyTorch and TensorFlow, where the quantum circuit outputs are interfaced with classical layers. Gradient computation for backpropagation is handled through PennyLane’s automatic differentiation, while model optimization is performed using the Adam optimizer, standard loss functions, and batch processing.

To ensure a fair and consistent comparison across architectures, all LSTM based models, including classical and quantum variants, were trained under identical hyperparameter settings. Each model was trained with 20 epochs, which serves as a baseline epoch count sufficient to achieve stable convergence without excessive overfitting. The network configuration consisted of 64 hidden units, tanh activation with LSTM, ReLU for dense layers, a dropout rate of 0.2, Adam optimizer, a learning rate of 0.001, and a batch size of 32. Early stopping was employed to avoid both overfitting and underfitting by monitoring validation performance during training.

This training strategy allows the models to iteratively adjust weights over multiple passes through the data, facilitating effective learning of long-term dependencies while maintaining generalization capability. Model performance was evaluated using accuracy, F1 score, recall, precision, AUC, and training time, enabling a comprehensive assessment of both predictive effectiveness and efficiency. The comparative results of all evaluated LSTM variants are summarized in Table 2, providing a basis for identifying most effective framework for infertility prediction in women using PCOS EHR dataset.

**Table 2:** Various LSTM models for training.

S. No.	Algorithms	Fertility rate dataset					
		Precision	Recall	Accuracy (%)	F1 Score	AUC	Time (s)
1.	LSTM	88.1	88.3	90	88.3	91	92
2.	Unilateral LSTM	90.3	89.8	92.1	89.8	93.8	84
3.	Bilateral LSTM	89.6	89.4	92.8	89.6	93.2	89
4.	Hierarchical- and Attention-based LSTM	89.5	89.7	90.3	89.5	92.4	90
5.	Convolutional LSTM	89.2	89.1	90.8	89.1	92	91
6.	LSTM Auto Encoder	87.1	87.4	90.4	87.3	90.6	87
7.	Grid LSTM	86.3	88.2	90.6	88.2	91.1	86
8.	Cross-Model and Associative LSTM	87.5	88.1	89.3	87.5	90.3	87
9.	Quantum LSTM	92.3	90.4	94.5	90.4	96.1	79

To evaluate the effectiveness of the proposed framework, performance metrics were compared with representative ML and DL approaches reported in PCOS studies. As shown in Table 1, existing methodologies predominantly rely on classical ML classifiers such as RF, SVM, AB and GB models, achieving accuracies in the range of 86 to 94% with AUC values typically below 93%. While some ensemble-based approaches report higher accuracy, they often depend on extensive feature engineering, static clinical data, and increased model complexity, which may limit scalability and generalization.

**Figure 2:**
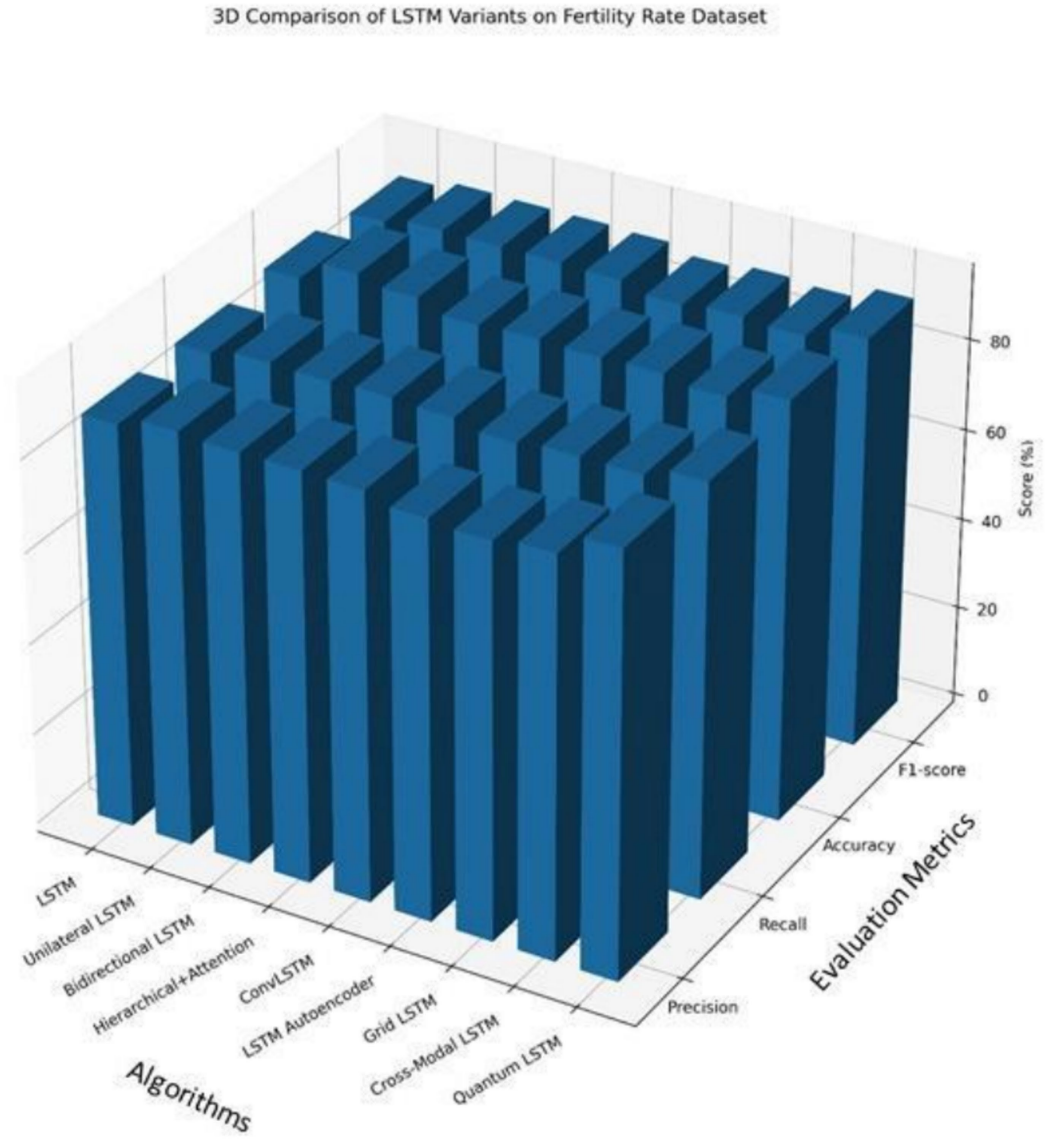
Various LSTM model performance visualization.

**Figure 3:**
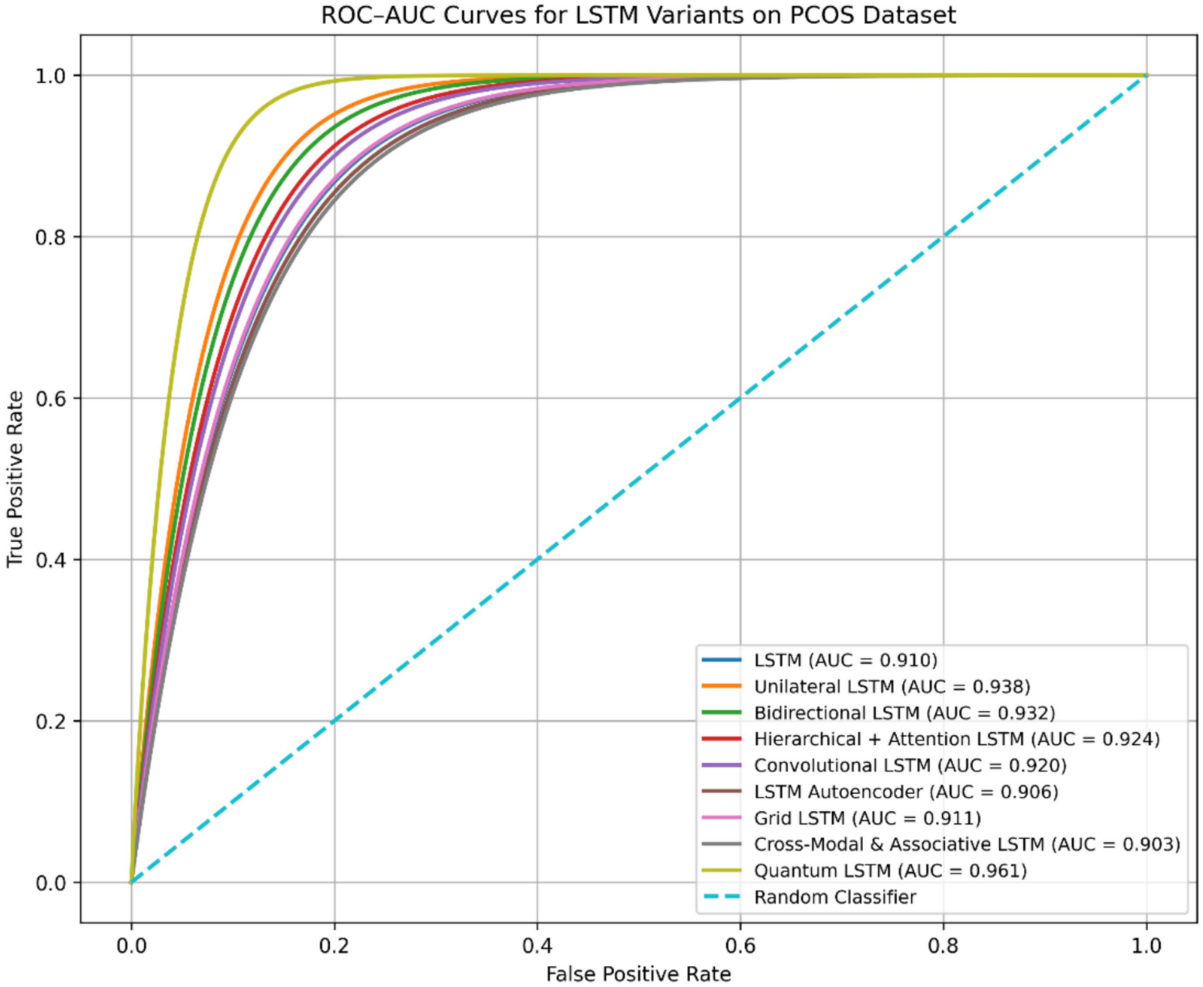
ROC–AUC curve of various LSTM models.

From the comparative analysis presented in Table 2 and Figure 2, it is evident that among all evaluated LSTM variants, the proposed quantum LSTM achieves the best overall performance on the PCOS dataset, attaining the highest classification accuracy of 94.5% while also requiring the lowest computational time of 79 s. The assessment of each model’s capability was further evaluated using the Area Under Receiver Operating Characteristic Curve (ROC–AUC). The ROC curves of representative LSTM models are illustrated in Figure 3.

Figure 3 shows Receiver Operating Characteristic (ROC) curves for various LSTM models comparing their ability to distinguish between PCOS and Non-PCOS class based on PCOS datasets. All models significantly outperform random classifier baseline, indicating effective learning of discriminative feature patterns. Among the evaluated approaches, quantum LSTM exhibits the highest AUC of 95.6%, followed by the unilateral LSTM with 93.8% and bidirectional LSTM with 93.2%. The AUC value of quantum LSTM indicates improved robustness and threshold independent performance, which is particularly important for early PCOS screening where sensitivity specificity trade-offs must be carefully balanced. Although various LSTM models achieve comparable accuracy, the ROC–AUC analysis demonstrates that the quantum LSTM maintains higher true positive rates at lower false positive rates conforming its superior discriminative capability.

**Figure 4:**
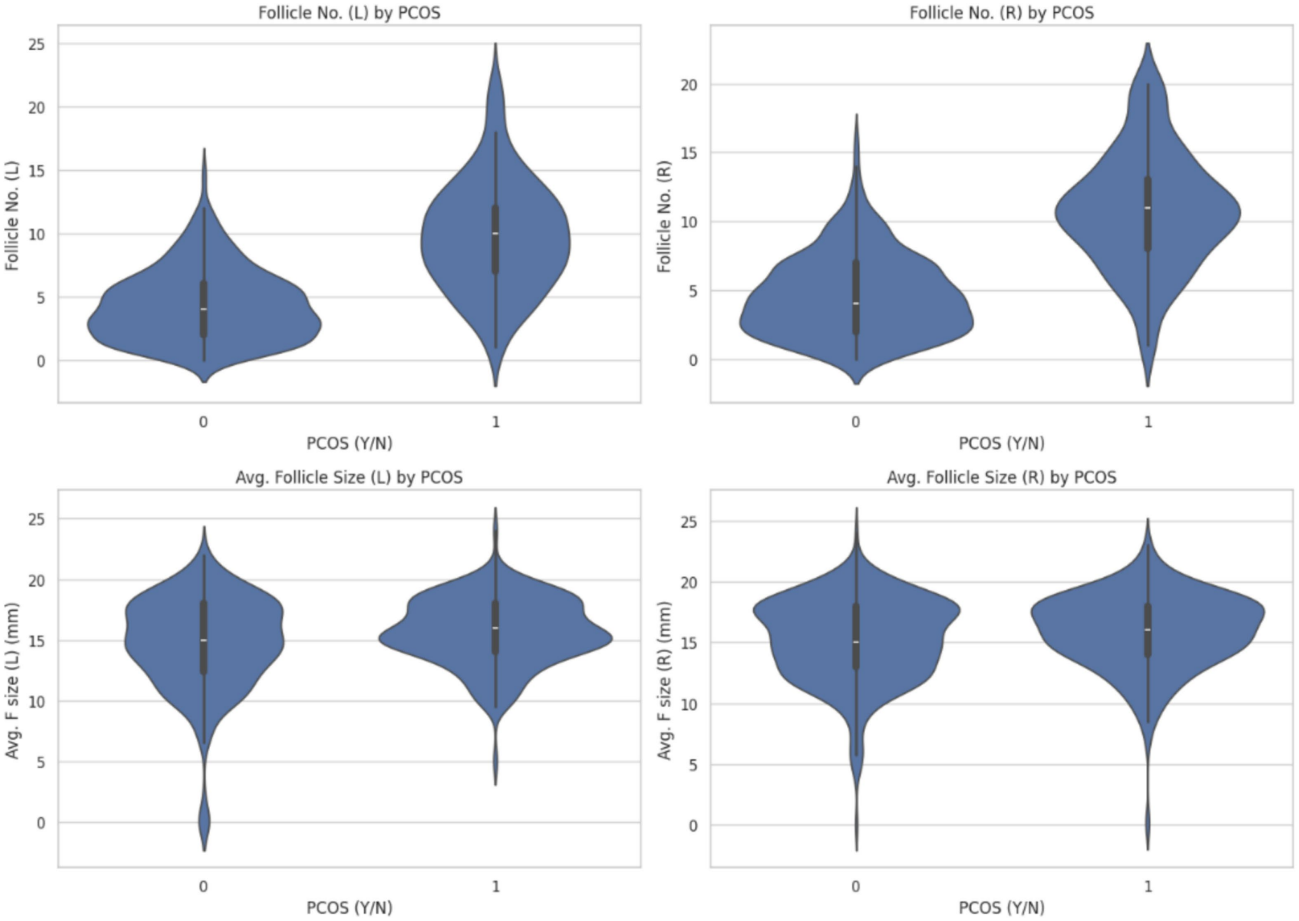
Ovarian follicle characteristics by PCOS diagnosis.

After training the dataset with various LSTM models, each algorithm was evaluated independently to examine the predicted behavior and feature influence at different stages of analysis. The resulting predictions are discussed below:

At first, the analysis reinforces the diagnostic criteria for PCOS, particularly the presence of multiple small follicles in the ovaries. These findings emphasize the relevance of ultrasound-based ovarian feature extraction in PCOS detection and support its integration into predictive modeling systems. The visualization is shown in Figure 4.

Figure 4 compares the number of follicles and the average follicle size in the left and right ovaries between individuals with and without PCOS.

- Follicle count (left and right ovaries): The upper plots clearly demonstrate that individuals with PCOS (label 1) tend to have more follicles in both the left and right ovaries compared to non-PCOS individuals. The distributions are wider and shifted upward, reflecting a larger follicular count, a hallmark feature of polycystic ovarian morphology.
- Average follicle size (left and right ovaries): The bottom plots show a relatively similar average follicle size across both the PCOS and non-PCOS groups, though the PCOS group exhibits greater variation and density concentration in the mid-to-upper size range. This suggests that while the number of follicles increases significantly in PCOS, their average size remains comparable to those without the condition.

**Figure 5:**
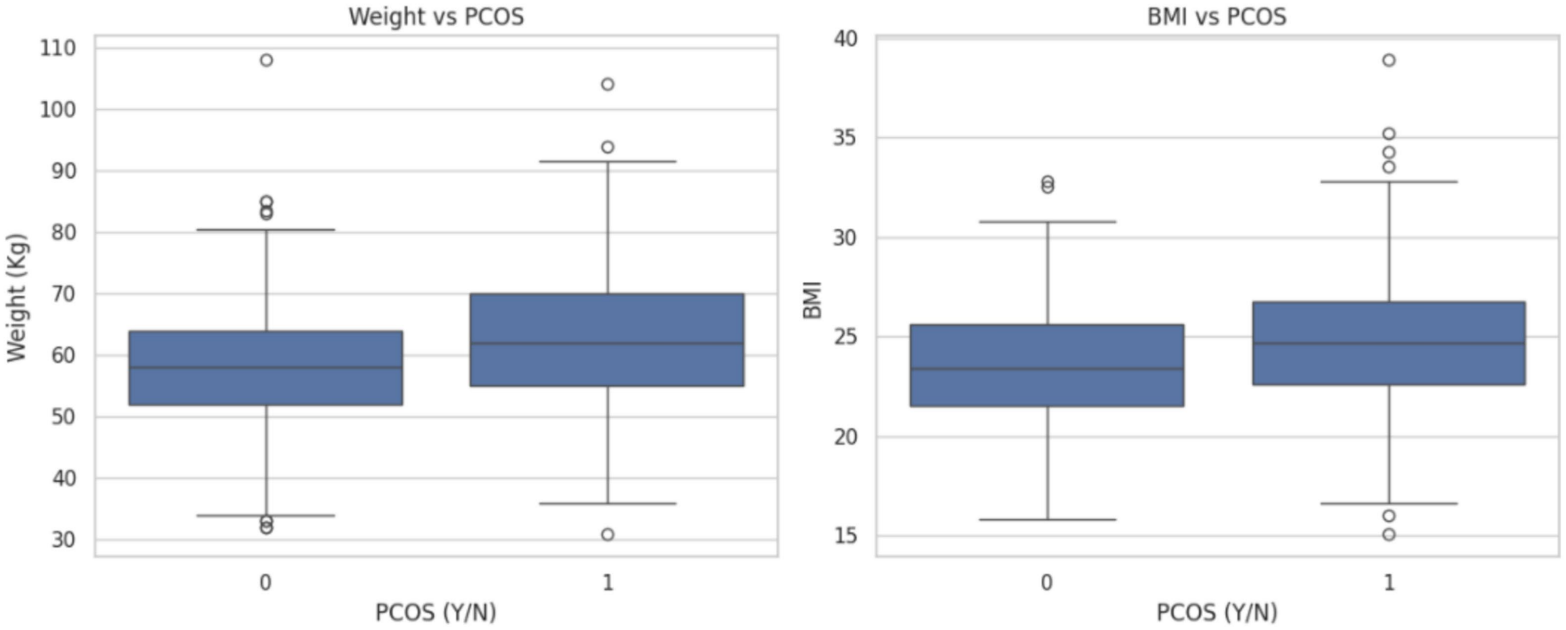
Graphs depicting weight and BMI distribution in relation to PCOS diagnosis.

Figure 5 depicts the potential association between increased body weight and BMI in the presence of PCOS for early prediction and diagnosis.

In Figure 5, the relationship between PCOS diagnosis (0 = No, 1 = Yes) and two physical health indicators: Weight (kg) and Body Mass Index (BMI) are observed as:

- Weight vs PCOS: Individuals diagnosed with PCOS (label 1) tend to have a higher median weight compared with those without PCOS (label 0). The spread of weights is also broader in the PCOS group, indicating greater variability. Outliers are observed in both groups, but the PCOS group shows several instances of higher weights.
- BMI vs PCOS: Similarly, the BMI distribution for individuals with PCOS shows a slightly higher median and a wider interquartile range compared with non-PCOS individuals. The presence of outliers in both groups highlights cases of particularly low or high BMI, but these are more concentrated in the PCOS group.

**Figure 6:**
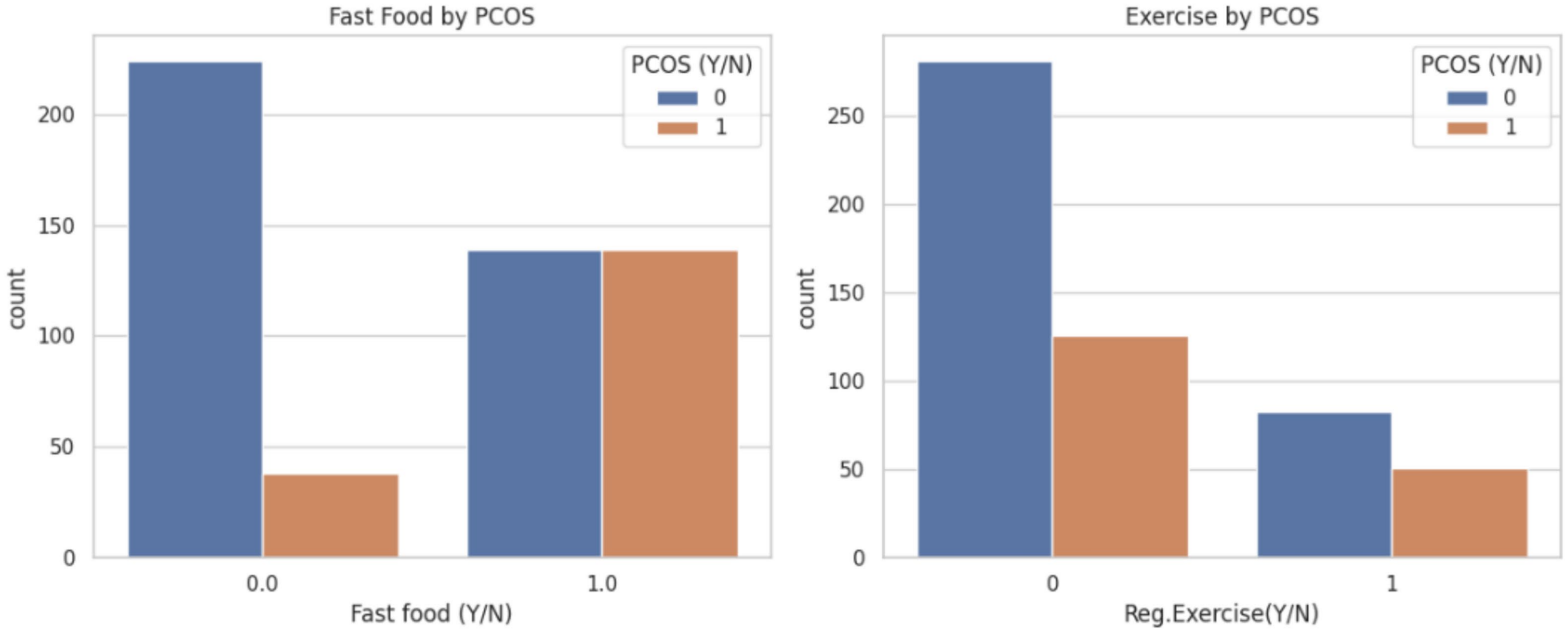
Lifestyle habits (fast food and regular exercise) on PCOS status.

Then visualized lifestyle factors, such as unhealthy diet and lack of physical activity, may contribute to or exacerbate PCOS symptoms. Thus, promoting regular exercise and reducing fast food intake may be beneficial preventive measures or adjunct strategies in managing PCOS. Figure 6 displays the visualization.

Figure 6 provides insights into the association between lifestyle habits like fast food consumption and regular exercise for PCOS diagnosis.

- Fast food on PCOS: A noticeably higher proportion of PCOS-positive individuals (orange bars, label 1) report regular consumption of fast food compared to non-PCOS individuals (blue bars, label 0). Conversely, a larger number of non-PCOS individuals abstain from fast food, suggesting a possible correlation between poor dietary habits and PCOS incidence.
- Exercise by PCOS: This shows a significant portion of PCOS-negative individuals engage in regular physical exercise, while a smaller percentage of PCOS-positive individuals do so. The lack of regular exercise appears more common in individuals diagnosed with PCOS.

**Figure 7:**
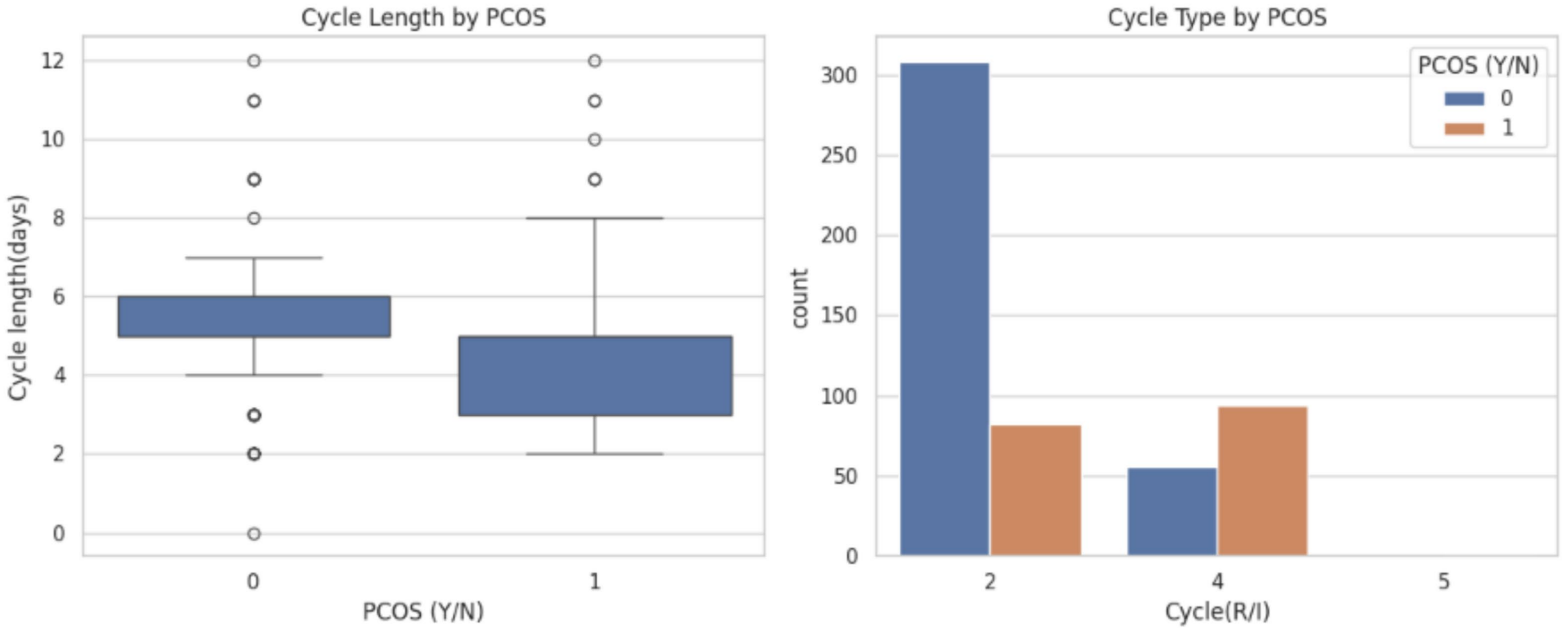
Menstrual cycle characteristics by PCOS status.

Later, visualized menstrual cycle irregularities in both length and pattern are strong indicators of PCOS because they can serve as key features in predictive modeling and early diagnosis frameworks. The visualization is shown in Figure 7.

Figure 7 highlights the differences in cycle length and cycle regularity between individuals with and without PCOS.

- Cycle length by PCOS: The plot reveals that individuals diagnosed with PCOS (label 1) tend to have more variable and shorter menstrual cycles compared with PCOS negative individuals (label 0). The presence of multiple outliers and a compressed interquartile range among PCOS-positive individuals indicates greater irregularity in menstrual cycle duration.
- Cycle type by PCOS: The bar chart compares the distribution of cycle types (e.g., 2: Regular, 4: Irregular, and 5: Unknown) between PCOS-positive and negative individuals. It is evident that regular cycles (type 2) are far more prevalent among non-PCOS individuals, while irregular cycles (type 4) dominate among PCOS cases. This pattern clearly supports the clinical understanding that menstrual irregularities are one of the most common symptoms of PCOS.

**Figure 8:**
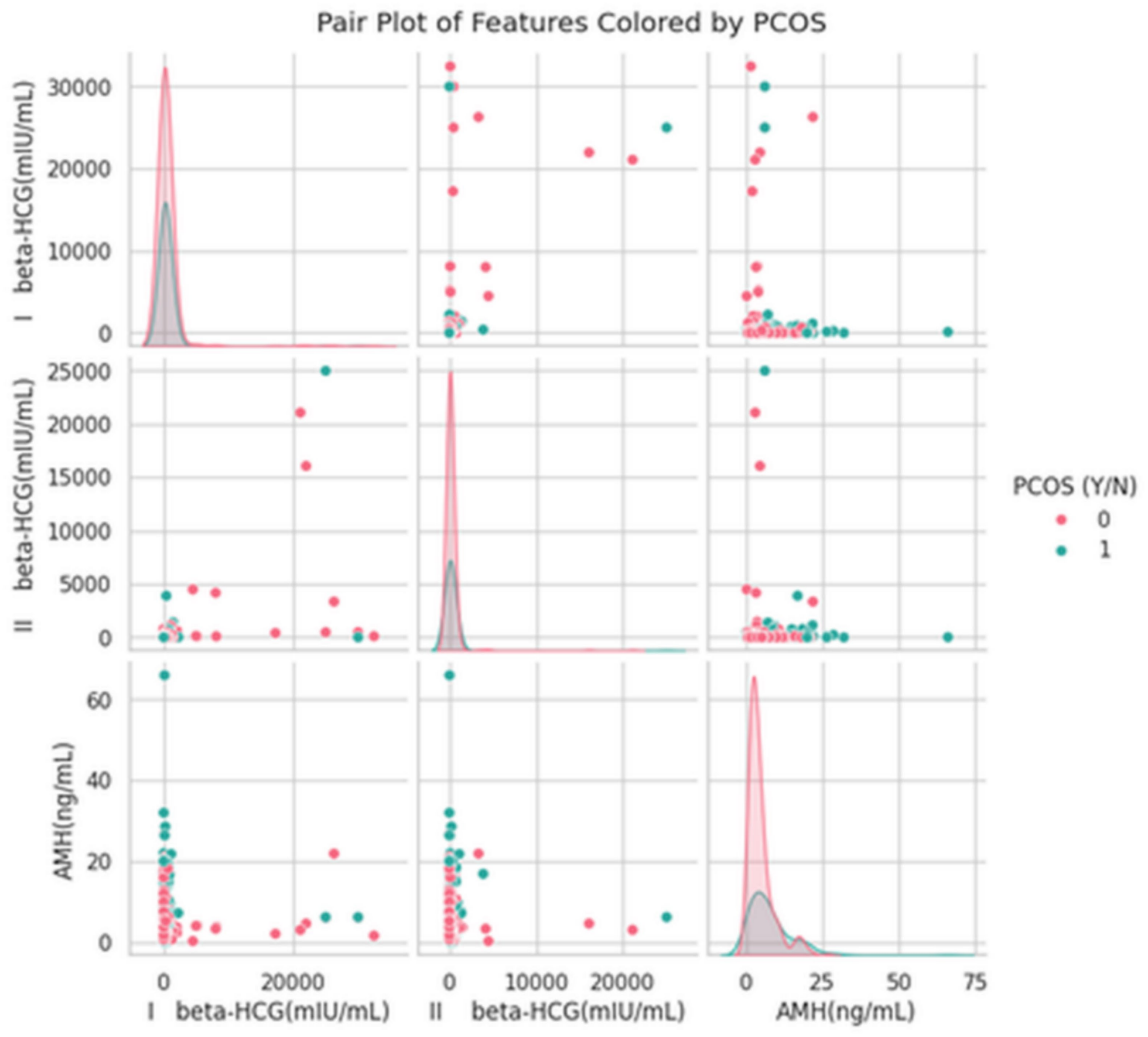
Relationship between hormonal markers and PCOS status.

Further, AMH serves as a discriminative feature in PCOS diagnosis, while beta-HCG values are visualized to analyze the hormonal changes based on the diagnostic models. The visualization is shown in Figure 8.

The Figure 8 visualizes the distribution and correlation between three key hormonal markers, like I beta-HCG, II beta-HCG, and Anti-Müllerian Hormone (AMH) across individuals with and without PCOS. Each point is colored based on PCOS diagnosis (0 = No PCOS, 1 = PCOS).

- I and II beta-HCG: The distributions of both I and II beta-HCG levels are highly right-skewed with a few extreme outliers, particularly in the non-PCOS group. The clustering of PCOS cases at lower beta-HCG levels suggests this hormone may have limited direct correlation with PCOS detection in this dataset, though aberrant peaks in non-PCOS cases may indicate outlier influence.
- AMH (Anti-Müllerian Hormone): A more distinct separation is observed in the AMH axis, with higher AMH levels predominantly appearing in PCOS cases. This aligns well with clinical evidence, as elevated AMH is a well-established biomarker for PCOS, reflecting increased antral follicle count.
- Inter-feature relationships: There is little to no linear correlation between beta-HCG and AMH, suggesting these markers provide independent hormonal insights in the context of PCOS.

**Figure 9:**
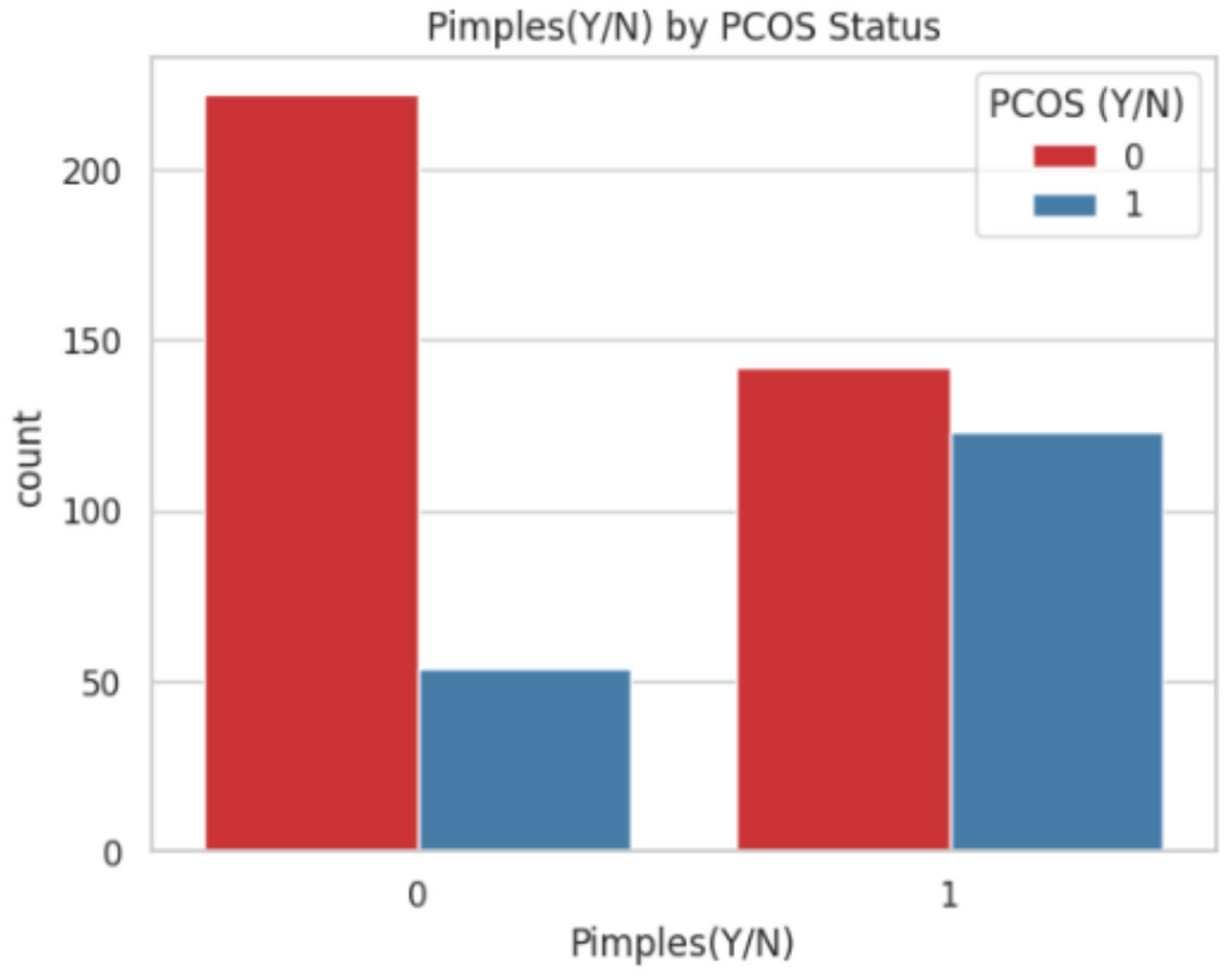
Frequency of pimples in PCOS and non-PCOS individuals.

The occurrence of pimples is visualized because it is more common among individuals with PCOS. While acne alone is not diagnostic, its co-occurrence with other symptoms (e.g., hirsutism, irregular cycles) makes it an important auxiliary feature in automated PCOS detection systems. The visualization is shown in Figure 9.

Figure 9 illustrates the distribution of pimples among individuals with and without PCOS. The *x*-axis represents the presence (1 = Yes) or absence (0 = No) of pimples, while the *y*-axis indicates the number of individuals. The red bars correspond to non-PCOS cases, reporting no pimples, though a sizable portion still experience them, indicating acne is not exclusive to PCOS and the blue bars corresponds to PCOS patients. Higher proportion of PCOS patients report having pimples, reinforcing the established association between hormonal imbalances in case of PCOS and increased acne occurrence.

This research builds and tests various LSTM based models for early detection of PCOS by combining clinical features, lifestyle variables, and hormonal markers. Out of all the architectures that were tested, the Quantum LSTM model achieved the highest accuracy (94.5%) with optimal precision and recall, and in less time, that is 79 s. Followed by the Unilateral LSTM with 92.1% accuracy and time of 84 s, while the other variants such as the Bidirectional, Convolutional, and Auto encoder LSTMs performed with low accuracy during training. In addition to the accuracy metric, the ROC-AUC analysis was performed to assess the capability of the model. The Quantum LSTM had the best AUC value (96.1%), showing that it was able to distinguish between PCOS and non-PCOS more effectively at different decision thresholds. Especially for early screening scenarios with significant sensitivity–specificity trade-offs, this improved ROC profile validates the proposed model’s resilience. Infertility in women caused by gynecological illnesses like PCOS can be better understood with the help of the LSTM-based temporal sequence models, which are well-suited to capturing intricate feature interactions and time-dependent patterns.

## 5 Conclusion

Infertility remains a growing global health concern, affecting nearly 15% of reproductive-age couples, with PCOS being one of the leading contributors to infertility in women. Early diagnosis and personalized intervention are essential to enhance reproductive outcomes and overall wellbeing. In this study, an AI-Quantum framework incorporating various LSTM and a Quantum LSTM models was developed to enable early detection of PCOS using EHR data. Among the evaluated models, the Quantum LSTM demonstrated superior performance, achieving high accuracy (94.5%) along with optimal precision, recall, and a reduced run time of 79 s. These results highlight the potential for aiding early diagnosis and fertility management by effectively leveraging critical clinical markers. The findings further emphasize the multifactorial nature of PCOS and its strong association with infertility. EHR-derived features, including anthropometric indicators (weight and BMI), lifestyle habits (fast food intake and lack of exercise), menstrual cycle characteristics, ovarian ultrasound findings, and hormonal markers (AMH and beta-HCG), proved highly informative in distinguishing PCOS-positive individuals. Additionally, clinical symptoms, such as hirsutism, hair loss, skin darkening, and pimples, reinforced the diagnostic value of the integrated feature sets. By leveraging EHR data and applying advanced temporal modeling, this proposed approach enhances PCOS detection and supports early intervention strategies, thereby reducing diagnostic delays and promoting preventive reproductive healthcare. The integration of an interpretable AI–Quantum framework with routine clinical data paves the way for scalable, real-time, and personalized diagnostic support systems in gynecology and fertility care. Despite these promising results obtained by proposed AI–Quantum hybrid framework, several limitations should be acknowledged, as the experimental evaluation was conducted on moderately sized dataset comprising 541 patient records with 44 features that are enough for preliminary validation, may limit the broader generalizability of the findings. Furthermore, the Quantum LSTM model was evaluated using a quantum simulation environment rather than physical quantum hardware and therefore the observed computational benefits should be interpreted as quantum inspired rather than definitive quantum advantage. Future work should focus on incorporating larger, multicenter, and longitudinal EHR datasets, as well as deploying the framework on emerging quantum hardware platforms to more rigorously assess scalability, disease progression modeling, and true quantum benefits in infertility predictions.

## Data Availability

The human samples used in this study were collected from dataset taken from publicly available Kaggle dataset: (https://www.kaggle.com/datasets/prasoonkottarathil/polycystic-ovary-syndrome-pcos/data).

## Footnotes

1
https://www.kaggle.com/datasets/prasoonkottarathil/polycystic-ovary-syndrome-pcos/data
